# Genomic breed prediction in New Zealand sheep

**DOI:** 10.1186/s12863-014-0092-9

**Published:** 2014-09-16

**Authors:** Ken G Dodds, Benoît Auvray, Sheryl-Anne N Newman, John C McEwan

**Affiliations:** AgResearch, Invermay Agricultural Centre, Private Bag, 50034 Mosgiel, New Zealand

**Keywords:** Breeds, Sheep, Prediction, Genomic selection

## Abstract

**Background:**

Two genetic marker-based methods are compared for use in breed prediction, using a New Zealand sheep resource. The methods were a genomic selection (GS) method, using genomic BLUP, and a regression method (Regp) using the allele frequencies estimated from a subset of purebred animals. Four breed proportions, Romney, Coopworth, Perendale and Texel, were predicted, using Illumina OvineSNP50 genotypes.

**Results:**

Both methods worked well with correlations of predicted proportions and recorded proportions ranging between 0.91 and 0.97 across methods and prediction breeds, except for the Regp method for Perendales, where the correlation was 0.85. The Regp method gives predictions that appear as a gradient (when viewed as the first few principal components of the genomic relatedness matrix), decreasing away from the breed centre. In contrast the GS method gives predictions dominated by the breeds of the closest relatives in the training set. Some Romneys appear close to the main Perendale group, which is why the Regp method worked less well for predicting Perendale proportion. The GS method works better than the Regp method when the breed groups do not form tight, distinct clusters, but is less robust to breed errors in the training set (for predicting relatives of those animals). Predictions were found to be similar to those obtained using STRUCTURE software, especially those using Regp. The methods appear to overpredict breed proportions in animals that are far removed from the training set. It is suggested that the training set should include animals spanning the range where predictions are made.

**Conclusions:**

Breeds can be predicted using either of the two methods investigated. The choice of method will depend on the structure of the breeds in the population. The use of genomic selection methodology for breed prediction appears promising. As applied, it worked well for predicting proportions in animals that were predominantly of the breed types present in the training set, or to put it another way, that were in the range of genetic diversity represented by the training set. Therefore, it would be advisable that the training set covered the breed diversity of where predictions will be made.

**Electronic supplementary material:**

The online version of this article (doi:10.1186/s12863-014-0092-9) contains supplementary material, which is available to authorized users.

## Background

Breed prediction is a useful tool for a number of reasons. Breed societies could use breed prediction to help audit registrations for authenticity. It may be of interest to determine the breed of commercial (unpedigreed) animals with desirable characteristics, for example from a slaughter facility. Alternatively, a breed description may be vague (e.g. a new ‘breed’, or descriptive, such as “meat composite”), and a better description of the contributing breeds is required. Within genomic selection (GS) programmes, breed prediction can be used for quality control of research and industry samples. This includes verifying the sample identification (a mis-identified sample could be revealed as a breed mismatch) and applying any breed rules relevant to the GS application (if the genomic prediction equations are to be applied to only some specific breeds).

Assigning observations to groups, where training data (known group membership) are available, is known as discriminant analysis in the statistics literature [1] or supervised learning in the machine learning literature [2]. A number of tools exist for predicting breed using genetic markers. In the ecological literature, these are often referred to as ‘assignment’ methods, and in general endeavour to assign an individual as belonging to one of a set of possible populations. They generally do not allow mixed (fractional) assignment, which is one of the goals of the present work.

A commonly used statistical method for assignment is (linear) discriminant analysis. Even if the goal was to assign an individual to a single population (rather than predict composition), these methods are not successful when there are many more predictors than observations for training [3]. This is due to ‘overfitting’ issues (the predictor fits the specific differences in the training set which are not representative of the populations).

Principal component analysis (PCA) is a multivariate statistical technique for summarising data from many variables into a few variables which explain as much of the variation in the data as possible. It is often used to investigate unknown clustering structure (i.e., cluster analysis or unsupervised clustering). While it does not use breed information directly, animals of the same breed tend to be located close together in plots of the leading principal components from a PCA analysis of single nucleotide polymorphisms (SNPs). This suggests that PCA might be a useful method for breed discrimination which does not suffer from the overfitting issue as for discriminant analysis. A drawback is that it does not easily translate into estimated breed proportions; its main use would be to verify that an animal is (or is not) the recorded breed.

A popular method for understanding population structure is the model-based clustering method implemented in the program STRUCTURE [4]. This method has been applied to surveys of breed variation in sheep [5] and cattle [6]. However, this method does not produce a prediction equation and requires a re-analysis when new data is added. Some alternative approaches are based on regression methods [7] and GS methods [8,9].

Our focus here is in methods that produce a prediction equation (i.e., a direct function of SNP genotypes), which can then be applied without further reference to training sets. We investigate two such methods in New Zealand sheep, one motivated by GS methods, and the other using a regression approach.

## Results

### Principal component analysis

**Figure 1:**
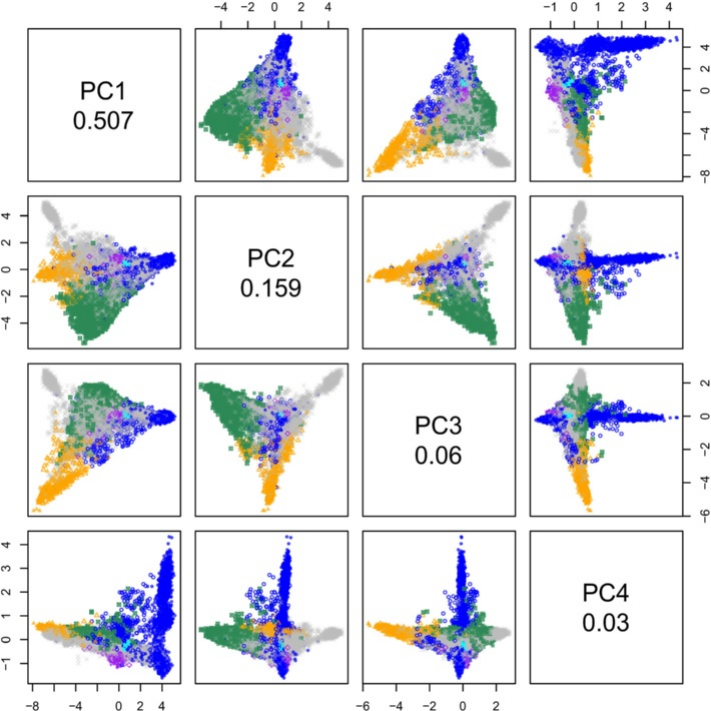
**The first four principal components of the genomic relationship matrix.** Scatterplot matrix of the first four principal components (PC1 to PC4) applied to the full dataset of 13,118 animals. Key: blue circle Romney; green square Coopworth; purple diamond Perendale; grey triangle Texel; X Other; where animals are coloured (other than grey) if more than 50% of that breed) and the symbols are filled if they are more than 90% of that breed. The proportion of variance explained by each of the components is shown on the diagonal.

**Figure 2:**
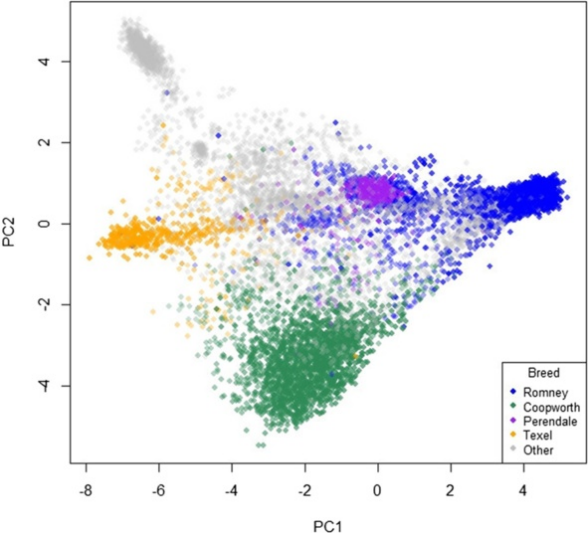
**The first two principal components of the genomic relationship matrix.** Plot of the first two principal components (PC2 v PC1) applied to the full dataset of 13,118 animals. Animals are coloured (other than grey) by their predominant breed with lighter shading for lower proportions of that predominant breed. Points are semi-transparent so that more intensely coloured regions correspond to regions where the total amount of that breed is high.

The first four principal components (PC1 to PC4) are illustrated in Figure 1. The analysis has been reasonably successful at separating out breeds, although the breed groupings are not completely distinct. The first principal component contains more than half (50.7%) of the variation in the relationship matrix, followed by 15.9%, 6.0% and 3.0% for the next three components. Therefore the first two components have been chosen to display results. Figure 2 shows these two components in more detail.

### Genomic selection method

**Figure 3:**
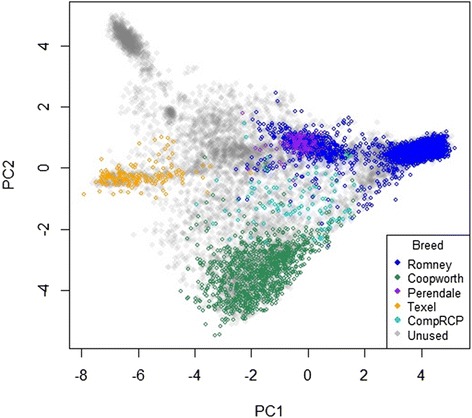
**Principal component plot of the training set.** Scatterplot matrix of the first two principal components (PC2 v PC1) of the training set (coloured) for the genomic selection method.

The principal components of the training set animals are shown in Figure 3. These fall mainly into four clusters corresponding to the four breeds used (although in these dimensions, Perendales appear to cluster with a subgroup of Romneys). The estimated heritabilities (from the estimated heritability analyses) were 0.89, 0.83, 0.86 and 0.87 for Romney, Coopworth, Perendale and Texel, respectively. These are lower than the value chosen for the fixed heritability analyses, suggesting more errors or more non-additivity due to genomic relationships than initially thought. Breed predictions from both methods (fixed and estimated heritability) were compared on the validation set. The regression of predictions from the fixed heritability method on those from the estimated heritability method gave correlations in excess of 0.998, slopes between 0.99 and 1.01, and intercepts between -0.001 and -0.002, i.e. the predictions were almost identical. Only the fixed heritability results, which required less computation, are presented in what follows.

Predicted breed proportions were regressed on recorded breed proportions in the validation set (subset of the October 2010 dataset born in or after 2008, and one of the breed types used in training). Results are shown in Table 1. The correlations were all high, and the regressions close to identity. Such correlations (sometimes divided by √h^2^ first) are what are usually reported as ‘realised’ accuracies in GS studies. Model-based accuracies for predicting each breed proportion were the same, as these do not depend on the variable being analysed (only on the heritability which was fixed at the same value, and the relationships between the animals).

**Table 1:** **Regression of recorded on predicted breed proportion (genomic selection method) in the October 2010 validation set**

**Breed (trait)**	**Correlation**	**Intercept**	**Slope**	**Mean accuracy** ^**a**^
Romney	0.985	−0.010	1.000	0.743
Coopworth	0.970	0.008	0.962	0.743
Perendale	0.971	−0.002	0.971	0.743
Texel	0.919	0.004	1.058	0.743

^a^Mean of the model-based accuracies.

### Comparison with other methods

**Figure 4:**
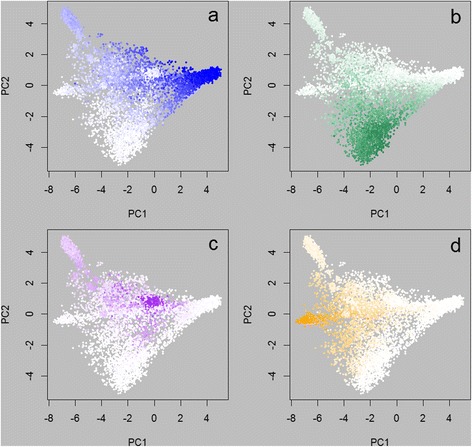
**Predicted breed proportions using the genomic selection method.** Predicted proportions of each of four breeds, using the genomic selection method, plotted on the positions of the first two principal components (PC2 v PC1) for the 8776 animals not in the training set. Colours range from white (proportion of zero) to a full intensity colour (proportion of one). Subpanels show the predicted proportions of **a)** Romney, **b)** Coopworth, **c)** Perendale, **d)** Texel.

**Figure 5:**
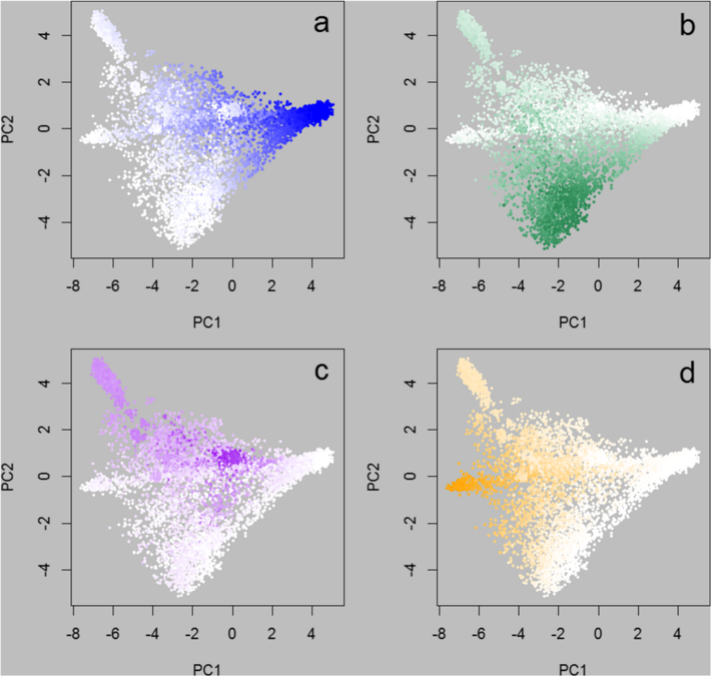
**Predicted breed proportions using the regression method.** Predicted proportions of each of four breeds, using the regression method, plotted on the positions of the first two principal components (PC2 v PC1) for the 8776 animals not in the genomic selection training set. Colours range from white (proportion of zero) to a full intensity colour (proportion of one). Subpanels show the predicted proportions of **a)** Romney, **b)** Coopworth, **c)** Perendale, **d)** Texel.

**Figure 6:**
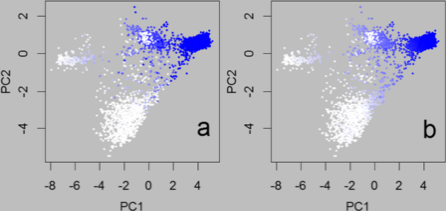
**Predicted Romney proportions for the training set.** Predicted Romney proportions, plotted on the positions of the first two principal components (PC2 v PC1) for the 4342 training animals available in the August 2011 dataset. Colours range from white (proportion of zero) to a full intensity blue (proportion of one). Subpanels show the two prediction methods: **a)** Genomic Selection, **b)** Regression.

**Figure 7:**
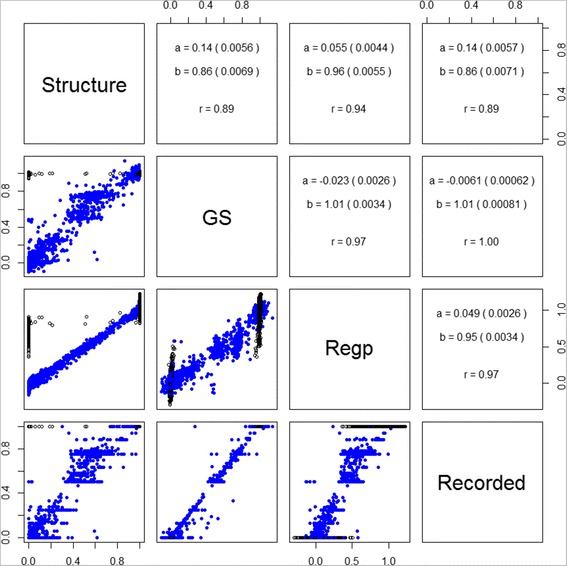
**Comparison of Romney breed predictions.** Scatterplots and regression summaries comparing four methods of obtaining Romney breed proportions (Recorded is from SIL; GS is genomic selection method; Regp is the regression method, Structure is from using STRUCTURE). STRUCTURE results are for all non-training and Romney training animals available in October 2010, while results for the other methods use the full dataset of 13,118 animals. Statistics in the upper panels refer to the intercept (a), slope (b) and correlation (r) for the regression of the y-axis on x-axis for the panel diagonally opposite, with standard errors given in parentheses.

The GS and Regp prediction equations were applied in the full dataset of 13,118 animals, while the STRUCTURE analysis included only the 4944 animals available in October 2010. Results for various subsets were investigated. Figures 4 and 5 show the predictions for the genomic selection method and regression method, respectively, applied to the subset of 8776 animals that were not part of the training set. Figure 6 shows the predicted proportions of Romney, using genomic selection and regression, for the subset of 4342 animals used for training. Comparisons between the methods are shown in Figure 7 for Romney predictions in all animals for which a method was applied. Table 2 shows the mean results from applying the equations to ‘purebred’ animals (that were not in the training set).

**Table 2:** **Predicted breed proportions in pure breed sets**

		**Genomic selection**				**Regression**			
**Breed**	**n**	**pRom**	**pCoop**	**pPere**	**pTex**	**pRom**	**pCoop**	**pPere**	**pTex**
Romney	1496	**0.985**	0.007	0.003	0.002	**0.996**	−0.003	0.003	0.003
Coopworth	286	0.022	**0.937**	0.011	0.021	−0.030	**0.979**	0.013	0.035
Perendale	262	0.036	0.017	**0.933**	0.009	0.031	0.008	**0.957**	0.003
Texel	57	0.025	0.041	0.037	**0.869**	−0.026	0.049	0.036	**0.938**
Corriedale	42	0.084	0.403	0.212	0.145	−0.032	0.417	0.344	0.250
Poll Dorset	39	0.333	0.090	0.099	0.054	0.044	0.227	0.452	0.241
Suffolk	25	0.241	0.127	0.318	0.140	0.061	0.193	0.467	0.249
Finnish Landrace	12	0.123	0.167	0.217	0.134	0.003	0.160	0.504	0.294
Marshall Romney	10	0.709	0.110	0.116	0.015	0.530	0.160	0.223	0.076
Wiltshire	7	0.182	0.351	0.255	0.070	0.061	0.381	0.388	0.151
Southdown	6	0.281	0.075	0.355	0.111	0.083	0.148	0.505	0.236
Dorper White	6	0.237	0.079	0.292	0.107	0.024	0.135	0.545	0.242
Cheviot	4	−0.444	−0.033	1.389	0.046	−0.447	0.039	1.293	0.105
Finn x Texel	4	0.249	0.139	0.100	0.396	0.182	0.119	0.217	0.468
Dorset Down	4	0.234	0.122	0.343	0.104	0.075	0.151	0.510	0.232
South Suffolk	4	0.227	0.073	0.378	0.137	0.051	0.155	0.518	0.244
Primera^a^	751	0.29	0.10	0.23	0.11	0.05	0.19	0.47	0.26
Highlander^a^	383	0.38	0.11	0.09	0.28	0.31	0.10	0.21	0.37

Mean predicted proportions, using each of the methods studied, for Romney (pRom), Coopworth (pCoop), Perendale (pPere) and Texel (pTex) in animals that are purebred (recorded as 100% of a particular breed; breeds with at least four animals available shown) and not used in the genomic selection training set. Proportions where the breed being predicted is the same as the recorded breed are shown in bold.

^a^These animals were not recorded on SIL at the time of analysis, but belonged to flocks with these breed designations.

## Discussion

### Breeds

Methods for predicting breed proportion have been applied using a set of SNP genotyped New Zealand sheep. This dataset was collected as part of a genomic selection research and development programme, with a focus on maternal breeds, which are the major proportion of New Zealand sheep. The dataset is not a survey of genetic material within New Zealand. Predictions were developed for the major breeds represented in this dataset – Romney, Coopworth, Perendale and Texel.

What is a breed? The concept of breeds can be vague [10,11]. In theory they are a homogeneous, closed breeding subpopulation. However, it is not clear when a new breed has been constituted, and a breed may arise formally (with a society and its associated rules), or informally (e.g. a group of farmers having the same breeding goals, swapping genetic material amongst themselves without external genetics being introduced). A breed society may allow ‘grading up’ or the infusion of a limited proportion of genetic material from other breeds (e.g. Coopworths). Therefore, some breeds may be genetically quite diverse, and some may consist of strains or lines. In both cases, an animal of the breed may be quite different, genetically, from the breed mean. In this report we have, where available, relied on the breeds as recorded on SIL. Therefore, the aim is to predict that recorded breed if it had been unknown.

### Breed prediction

The methods used do not explicitly account for the compositional nature of the data (i.e. that the breed proportions are values between zero and one and sum to one). Despite this, most of the predicted proportions do lie near or within this range (Table 3). In particular, at most 0.21% of genomic selection breed predictions deviate by more than 0.1 from the feasible range (Table 3). The figures that use colour intensity to portray breed proportion have thresholds at zero and one, so that values ≤0 are shown as white, and values ≥1 are shown as the full intensity colour.

**Table 3:** **Ranges of breed proportion predictions**

		**Number of observations in range**					**%**
**Method**	**Breed**	**≤ − 0.1**	**(−0.1,0]**	**(0,1]**	**(1,1.1]**	**>1.1**	**≤ − 0.1 or >1.1**
GS	Romney	23	836	10735	1519	5	0.21
GS	Coopworth	2	2155	10751	206	4	0.05
GS	Perendale	0	3693	9347	74	4	0.03
GS	Texel	4	4096	9014	4	0	0.03
Regp	Romney	326	1575	8969	1737	511	6.38
Regp	Coopworth	12	2779	9742	391	194	1.57
Regp	Perendale	72	2875	9901	167	103	1.33
Regp	Texel	0	3773	9286	54	5	0.04

Number of breed proportion predictions in different ranges when applied to the full dataset (13,118 animals), using genomic selection (GS) and regression (Regp).

Use of the 50 k SNP chip data has allowed good predictions of breed. This was seen for the GS method where correlations between predicted proportion and recorded proportion in a validation set were high. It is also evident from Figures 4 and 5, and Table 2, where estimated proportions of a breed for purebred animals (non-training) of that breed ranged from 0.869 to 0.996 (four breeds, two methods), with estimates for non-contributing breeds (e.g. the proportion of Coopworth for purebred Romneys) ranging from −0.03 to 0.05. All predictions were performed with the full set of markers; Frkonja *et al.* [9] found that prediction accuracy did not deteriorate appreciably when reducing the number of SNP used from 40,000 to 4,000 equally spaced SNPs when using several prediction methods.

The methods have, however, often predicted sizeable proportions of a prediction breed in animals which are purebred for a breed not considered here for prediction (Table 2). In some cases this reflects breed development, for example the prediction proportion of Romney is high in Marshall Romneys. The latter breed has been developed as a subpopulation of Romneys [12], so this is not surprising. Similarly Kuehn *et al.* [7] found it harder to distinguish Angus and Red Angus cattle than the other breed comparisons they considered. A reverse example is seen for the prediction in four Cheviots, which are estimated as being approximately 130% Perendale and −45% Romney. The Perendale was developed as a Cheviot/Romney cross, so algebraically, if Perendale = ½(Cheviot + Romney) then Cheviot = 2 × Perendale – Romney, not too dissimilar to the result we obtained, considering that the Perendale is likely to have changed from its foundation. This does illustrate that care needs to be taken when interpreting the predicted breed proportions.

### Prediction in other breeds or groups

**Figure 8:**
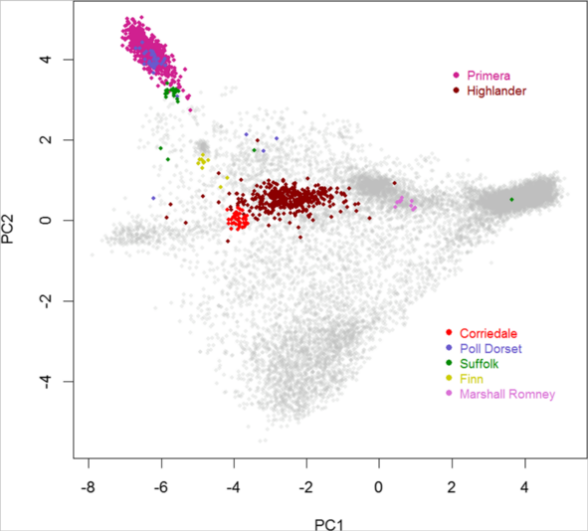
**Non-training pure breed animals.** Plot of the first two principal components (PC2 v PC1) of the full set of 13,118 animals, with non-training pure breed animals, with at least 10 animals per breed, highlighted.

An aspect where the prediction methods don’t perform so well is that many of the other (non-prediction) breeds appear to have a moderate proportion of the prediction breeds. For example the most prevalent non-prediction pure breeds are Corriedales which appear as ~ 40% Coopworth, and Poll Dorsets as ~ 30% Romney (GS method or 40% Perendale + 20% Coopworth + 20% Texel (Regp method)). The GS method appears to give moderate Romney predictions and the Regp method moderate Perendale predictions, for many of the breeds. These results may be due in part to some shared ancestry or they may reflect the inability to distinguish breeds which have not contributed to the training set or prediction method (in the case of Regp). The difference between the GS and Regp methods for this situation reflects their properties as discussed below. The first two PCs for breeds with at least 10 pure breed animals genotyped are plotted in Figure 8. Most of these breeds cluster tightly, although Poll Dorset and Suffolk have a few members which plot away from their main groups. This may represent mis-recording (seems quite likely for the Suffolk which plots within the main Romney cluster), or may be an artefact of how the animals were sampled for the R&D programme. If they are mis-recordings, they would inflate the predicted breed proportions by only a few%.

The prediction equations were also applied to two groups of animals without SIL recorded information at the time of analysis (Table 2, Figure 8). These are two recent breed developments (Highlander and Primera) by Rissington Breedline Ltd (http://www.focusgenetics.com/). Highlanders, described as a Romney, Texel, Finn cross, appear to be ~ 40% Romney, 25% Texel, 10% Coopworth, 10% Perendale. GS and Regp give somewhat different predictions for Primeras; ~30% Romney, 20% Perendale, 10% Coopworth, 10% Texel using GS, but a minor Romney component and ~ 45% Perendale, 25% Texel and 20% Coopworth using Regp. They are a terminal breed and have considerable Dorset and Suffolk type ancestry. They are not described as having any Texel component.

In the New Zealand case described in this work, the animals utilised were derived from industry genomic selection breeding programmes. The results show a need in breed prediction to include a wider range of breeds present in New Zealand including: Finn, East Friesian, Corriedale, Merino, Suffolk and Dorset. It would also be beneficial to examine the performance of these predictions in suitable overseas breed samples from the Ovine HapMap project [5] and perhaps include some of those results in the training set. This work is currently underway.

### Comparison of methods

The prediction methods give similar results, but there are some differences. Comparisons between the methods are shown in Figure 7 for Romney predictions in all animals, and for the training and non-training subsets in Table 4 (all prediction breeds). The GS method gives almost identically the recorded breed proportion for the animals used in training (correlations are all > 0.999). The corresponding correlations are lower for the Regp method (0.94-0.97), and, apart from Perendale predictions, not much better than predictions (of recorded breed) in non-training animals. STRUCTURE uses a different output for training individuals compared to validation individuals, so that a breed probability can only be obtained for the specified breed. Therefore, the only training animals shown in the ‘Structure’ panels of Figure 7 are those recorded as (100%) Romney. A reasonable proportion of these are given in the STRUCTURE output as having low probability of being Romney. These are generally given Romney probabilities of 0.5-0.9 by the Regp method, lower than those for the training animals where the STRUCTURE Romney probability was high. The set of training animals given low Romney probabilities differed with the set of SNPs used. In contrast, the Romney probabilities for validation animals were consistent with regard to the set of SNPs used, and were very similar to those given by the Regp method. These results were similar when considering prediction of other breed proportions (data not shown). The almost perfect back predictions for GS can be expected, as this is a BLUP procedure for a ‘trait’ with very high heritability (0.95). Therefore most of the information for an individual used in the training set will come from that individual itself. As the heritability was set lower than 1, relatives (as determined by genomic relationships) will contribute some information, and this might explain why the regression slopes are close to 1 (if only the individuals themselves contributed, the regression of predictions on recorded values, i.e. opposite to that shown in Table 4, would have slope equal to the heritability).

**Table 4:** **Comparison of breed prediction results**

				**Intercept**		**Slope**		
**Set**	**Breed**	**Method1**	**Method2**	**Value**	**SE**	**Value**	**SE**	**Correlation**
Train	Romney	Recorded	GS	−0.005	0.000	1.008	0.000	1.000
Train	Romney	Recorded	Regp	0.056	0.003	0.939	0.004	0.968
Train	Romney	Regp	GS	−0.024	0.003	1.008	0.004	0.970
Train	Coopworth	Recorded	GS	−0.002	0.000	1.010	0.000	1.000
Train	Coopworth	Recorded	Regp	−0.002	0.002	0.949	0.003	0.974
Train	Coopworth	Regp	GS	0.012	0.001	1.013	0.003	0.976
Train	Perendale	Recorded	GS	0.000	0.000	1.010	0.000	1.000
Train	Perendale	Recorded	Regp	−0.016	0.001	0.919	0.005	0.945
Train	Perendale	Regp	GS	0.025	0.001	0.986	0.005	0.948
Train	Texel	Recorded	GS	0.000	0.000	1.022	0.000	1.000
Train	Texel	Recorded	Regp	−0.011	0.001	0.907	0.005	0.945
Train	Texel	Regp	GS	0.017	0.001	1.012	0.005	0.949
Non-train	Romney	Recorded	GS	−0.047	0.003	1.026	0.004	0.966
Non-train	Romney	Recorded	Regp	0.006	0.002	0.967	0.004	0.967
Non-train	Romney	Regp	GS	−0.079	0.002	1.049	0.003	0.971
Non-train	Coopworth	Recorded	GS	−0.026	0.002	1.007	0.004	0.964
Non-train	Coopworth	Recorded	Regp	−0.023	0.002	0.952	0.004	0.958
Non-train	Coopworth	Regp	GS	0.017	0.001	1.009	0.002	0.981
Non-train	Perendale	Recorded	GS	−0.020	0.002	0.951	0.006	0.925
Non-train	Perendale	Recorded	Regp	−0.041	0.002	0.819	0.008	0.849
Non-train	Perendale	Regp	GS	0.066	0.001	1.019	0.004	0.931
Non-train	Texel	Recorded	GS	−0.009	0.001	0.989	0.005	0.940
Non-train	Texel	Recorded	Regp	−0.027	0.002	0.868	0.006	0.913
Non-train	Texel	Regp	GS	0.037	0.001	1.080	0.004	0.954

Summary of regressions of breed proportions from Method1 on those from Method2 (Recorded is from SIL; GS is the genomic selection method; Regp is the regression method). Regression parameters shown are the intercept and slope, along with their standard errors (SEs), and the correlation. The sets are either the subset of training animals (Train), or the subset that were not training animals (Non-train).

The set of breeds in the training set is likely to have had some influence on the performance of the methods. Even though the different breeds are estimated from different analyses with the GS method, the inclusion of animals of other breeds is required to give variation in the response variable (breed proportion). The particular breeds were chosen as they were the predominant breeds, and have been the focus of the genomic selection R&D programme. Including animals of other breeds in the training sets may give lower Romney, Coopworth, Perendale and Texel predictions in breeds such as Dorset and Suffolk breeds, or the Primera composite, which are removed from the space occupied by the training animals used here. Using STRUCTURE in the manner used here requires a set of purebred training animals from each breed, although in principle it could discover groups if they are sufficiently distinct from the training breeds. For the Regp method, there needs to sufficient purebred animals of a breed to give good breed specific allele frequency estimates, before including that breed in the regression equations. Alternatively, methods such as least squares or a logistic regression approach [7] could be used to estimate purebred allele frequencies from a dataset including mixed-breed animals, thereby increasing the effective number of animals for a breed. Frkonja *et al.* [9] found that predictions remained good despite reducing the training set from approximately 100 per breed to 10 per breed. This suggests that good predictions could be obtained with fewer resources than used in the present study, although the minimum requirements (training set size and number of SNPs used) for any particular application will depend on the nature of the breeds involved and the accuracy of prediction required.

For each of the breeds being predicted, there are training animals that are predicted to have a breed proportion very near to 0 or 1 with the GS method, but not so close to these values using the Regp method. As has already been indicated, the GS results for training animals are very close to the recorded values, so these are mainly animals that are recorded as pure breeds for that breed, or do not contain the breed at all. For example (Figure 7), there is a set of training animals with Romney predictions that are high (>0.95) using genomic selection, but only moderate (between 0.5 and 0.7) using regression. These are mostly animals with PC1 between 0.5 and 1.5 and PC2 between 0 and 1. These are Romneys that plot very close to the Perendales on the PCA plot (e.g. Figure 2). In Figure 6a they appear as intensely coloured points, whereas in Figure 6b they are less intensely coloured. Figure 6a has a more speckled appearance than Figure 6b. These observations reflect the fact that the regression method uses the central position of a breed (as determined by the allele frequencies used) as its reference and it is the relative distances in this space that determine the estimated breed proportions (giving the appearance of a gradient in Figure 6b). As a consequence, the regression method will be more robust against having training animals with an incorrectly recorded breed. However it will not perform so well for breed distributions that are not spherically clustered as we have seen here with the Romneys stretching across the Perendale locations (in PC1-PC2 space). Regp tended to predict moderate contributions of Perendale in the non-prediction breeds (Table 2), perhaps because Perendale is reasonably central in PC1-PC2 space. GS tended to predict moderate proportions of Romney in the non-prediction breeds, perhaps because Romney was the dominant breed and had a few animals spread across the distribution of animals.

The gBLUP methods used in genomic selection studies will have similar properties to what was seen with GS here, i.e. predictions will be dominated by the closest (genomic) relatives in the training set [13], but might not be robust against gross data errors. However, such errors are less likely for quantitative traits than a simple recording trait that relies on parentage (as is the case for breed). As mentioned above, our goal here is to predict the recorded breed, and because many of the breeds do not form a tight cluster, the GS method would be preferable to the Regp method. On average the Regp method performed better in purebreds though (first four rows of Table 2).

It is interesting that the correlations given in Table 1 are much higher than the mean model-based accuracies. Correlations (possibly divided by √h^2^ to estimate correlation with true breeding value) in validation sets and model-based accuracies are both used as accuracy measures in genomic selection studies. Perhaps this is because the model accuracy is based on average inheritance (animal model) rather than a gametic inheritance model, and genomic relationships estimated true genomic relationships rather than expected genomic relationships. Another factor may be that the trait here is non-normal (constrained to the interval [0,1] with many animals at the boundaries of this interval), and the model-based method assumes normality.

### Detection of breed errors

**Figure 9:**
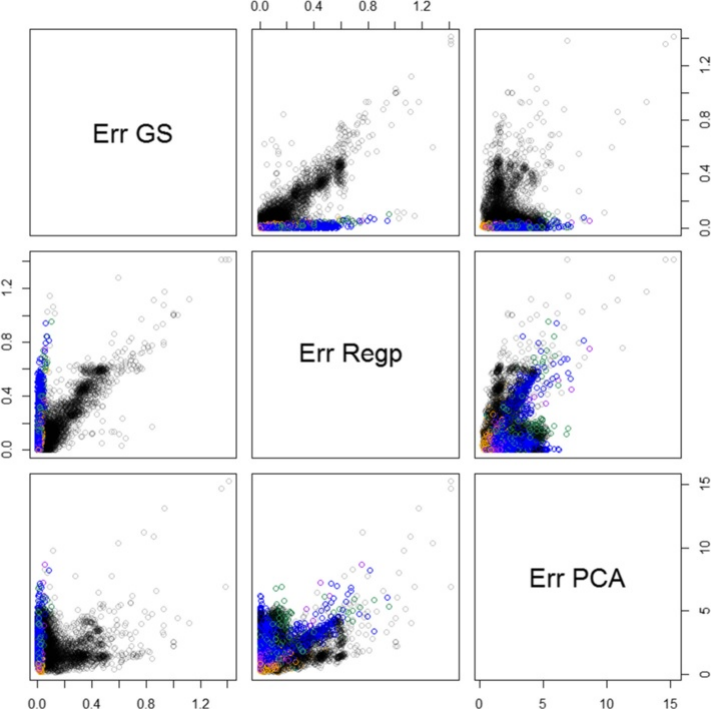
**Deviation from recorded breed.** Scatterplots of metrics of deviation from recorded breed based on genomic selection predictions (Err GS), regression predictions (Err Reg) and distances using the first 10 principal components (Err PCA) for the full dataset of 13,118 animals. Animals in the training set are shown in colour (blue cricle Romney; green circle Coopworth; violet circle Perendale; orange circle Texel; blue green circle CompRCP).

The metrics of deviation from recorded breed were calculated for the full set of 13,118 animals. For the PCA method, the first 10 components explained 83% of the variation. The metrics are shown in Figure 9. Animals which were recorded as one of the prediction breeds, and which had *Err*_*GS*_ >1 or *Err*_*Reg*_ >1 or *Err*_*PCA*_ >10 from an initial analysis were removed from the training data. As noted previously, the GS method predicts recorded breed almost perfectly for animals in the training set, and therefore is unlikely to be useful for diagnosing breed errors in the training set.

For the non-training animals, the error metrics are reasonably consistent between methods, and therefore tend to identify the same animals as outliers. The methods had previously been run before some particular animals had been removed for quality control reasons such as a breed mismatch. Predicted breed proportions in the vast majority of non-training were very similar to the updated predictions, but there were a few animals where GS predictions of a breed changed by up to 0.45.

Frkonja *et al.* [9] have also investigated genomic selection methods for the prediction of breed composition in cattle. They found that GS predictions using ~40,000 SNPs had correlations with pedigree ranging from 0.93 to 0.98, depending on which GS method was used, in an admixed population derived from two founding breeds. Frkonja *et al.* [9] found that STRUCTURE [4] performed similarly to the best GS method investigated (BayesB). Kuehn *et al.* [7] used both the regression method and Mendel [14] software to estimate breed proportions using ~50,000 SNPs in crosses between up to eight breeds. Both methods gave correlations with recorded breed composition of around 0.94.

As with these previous studies, we have used correlation (and regression) with recorded pedigree to assess a prediction method. However the actual breed composition in a crossbred animal will differ from its pedigree (average across ancestors) due to Mendelian sampling. Sölkner *et al.* [15] demonstrate this in a sheep crossbred population where the founders were also genotyped. The STRUCTURE program gave better predictions of breed proportion, as estimated by identity by descent methods (tracking chromosomal segments) than did pedigree. Therefore, the correlations quoted here (and in some other studies) are likely to be lower than the correlations with true genomic proportions. This is not relevant when breed prediction is for quality control with recorded breed, but is of interest for other applications.

The gBLUP method has also been used by VanRaden *et al.* [8], who applied it to estimating breed proportion using ~50,000 SNPs in validation sets of pure-breed dairy cattle. Averaged breed predictions were within 0.01 (proportion) of the stated breed. The results were able to discover an incorrect historic pedigree record (involving incorrect breed) of an ancestor with many descendants in the dataset. VanRaden *et al.* [8] suggest that the extremely good performance of breed prediction may be due to the large training set and that predictions were mainly tested in purebred animals. The latter would make predictions easier as there would be no difference between genomic and recorded breed proportions as noted for crossbreds above. In addition, it is difficult to compare methods across studies, as they use different breed sets with differing amounts of separation.

We would expect the gBLUP method to perform as well as other GS methods, as previous studies [16] have shown that genomic predictions with gBLUP are competitive for predicting ‘traits’ that behave as polygenic, which is expected for genomic breed proportions. It is interesting to note that the breed ‘phenotypes’ we have used do not satisfy the normal assumptions for BLUP methods. Specifically, an animal’s breed phenotype is exactly the mean of its parents’ breed phenotypes. If these are taken as imperfect measures of genomic breed proportions, then the error in the phenotype is correlated with the Mendelian sampling term. When we applied the gBLUP method to a cleaned dataset, and allowed genetic parameters to be estimated, the residual component was estimated at its lower bound (10^−8^). This may be a result of model inadequacy. Nevertheless, as we have shown here, using a high fixed heritability (0.95) appears to be a useful strategy for obtaining genomic breed predictions using gBLUP.

## Conclusions

Two methods that produce prediction equations have been examined for their utility in predicting breed composition in New Zealand sheep. In validation populations, both methods were found to be useful for predicting breed and generally gave similar predictions to STRUCTURE. The methods have strengths and weaknesses, but, in particular, the use of genomic selection methodology appears promising. As applied, it worked well for predicting proportions in animals that were predominantly of the breed types present in the training set, or to put it another way, that were in the range of genetic diversity represented by the training set. Therefore, it would be advisable that the training set covered the breed diversity of where predictions will be made.

## Methods

### Animals and genotypes

**Figure 10:**
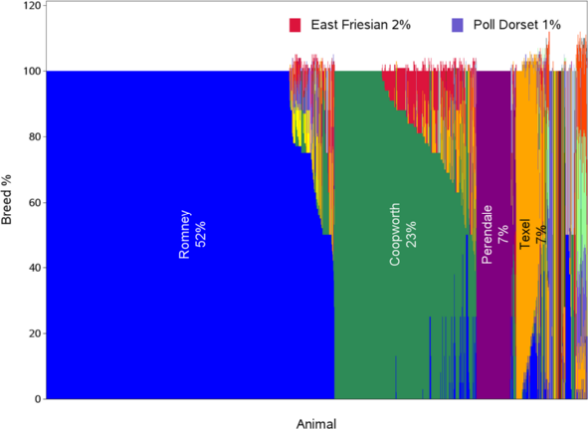
**Recorded breed composition.** SIL-recorded breed composition of the 8,705 SIL recorded animals. Each breed is represented by a different colour, but only the main breeds are identified. Rounding can result in breed percentages greater than 100%.

The animals used in this study were sheep from New Zealand that were sampled as a resource for GS, as described in Auvray *et al.* [17]. The 13,118 samples (9679 males and 3439 females) from animals born between 1986 and 2010 (Additional file 1: Figure S1) that were genotyped with the Illumina OvineSNP50 Beadchip until August 2011 were included. Breed information was extracted from the Sheep Improvement Limited (SIL; www.sil.co.nz) database for SIL flocks whose information was made available for GS studies. There were 8,705 animals with both genotype and recorded breed information. The animals were predominantly Romney, Coopworth, Perendale or Texel, but other breeds and various breed crosses were also present (see Figure 10 and Additional file 2: Table S1). SIL records an animal’s breed composition by averaging the parents’ breed components, but only allowing up to five different breed components, and applies a rounding up procedure which can result in the recorded breed percentage exceeding 100%. When parents are not on the database, breed can be supplied by the owner or taken as the breed designation of the birth flock. The data from 70 samples were removed before final analysis due to their identification being in doubt, either because their genotypes were almost identical to those of another sample (59 samples removed) or due to an obvious mismatch with recorded breed, using methods as described here. This article was approved for publication by AgResearch Ltd.

Genotype data were quality control (QC) checked [17]. Any SNPs that were discarded as part of the ovine HapMap (www.sheephapmap.org) project were removed, as were any SNPs with a call rate less than 0.97, an Illumina quality score (GC10) value less than 0.422, were not autosomal or that were monomorphic. Samples were QC checked by checking for consistency with duplicates, gender, parentage and breed.

### Genomic selection prediction equations for breed proportions

Genomic selection (GS) methods were applied to the recorded breed proportions (as ‘trait’ values) to develop predictions for breed proportions. Animals were chosen for training the prediction equations from the 5530 that were available in October 2010. Animals were assigned to breed groups if they had a recorded breed composition having more than 50% of either Romney, Coopworth, Perendale or Texel, or more than 50% of Romney, Coopworth or Perendale combined (hereafter denoted “CompRCP”). There were 4917 animals assigned to these breed groups. These were the main breeds represented in the resource, and were the breeds that were the focus of the GS programme. Animals from these breed groups and born prior to 2008 were chosen for training. There were 2840 Romneys, 1001 Coopworths, 288 Perendales, 164 Texels and 101 CompRCPs using these criteria. All these animals were used in training no matter what breed proportion was being modelled. There were 47,291 SNPs available after QC procedures were applied and these had an average call rate of 99.93%.

Prediction equations were calculated using the ‘gBLUP’ method, generally following the methods in Auvray *et al.* [17]. Four variables (proportion of Romney, Coopworth, Perendale or Texel; hereafter referred to as the ‘prediction breeds’) were fitted, one at a time, to an animal model with the numerator relationship matrix replaced by a genomic relationship matrix. For example

$$ {\mathbf{p}}_{\mathrm{Rom}}\sim \mathbf{1}\mu +\mathbf{Z}\mathbf{u}+\mathbf{e} $$

where **p**_Rom_ is a vector of recorded Romney proportions (ranging from zero to one) for the animals in the training set, **1** is a vector of 1’s of the same length, μ is a constant, **u** is a vector of molecular breeding values (mBVs) for each animal, **Z** is the incidence matrix (relates training set observations to animals) and **e** is a vector of residuals. The mBVs are modelled as a random effect with Var(**u**) = **G**_1_${\sigma}_a^2$, Var(**e**) = **I**${\sigma}_e^2$, Cov(**u**,**e**) = **0**, where **G**_1_ is the genomic relationship matrix (square with dimensions number of animals) using the first method described by VanRaden [18], namely

$$ {\boldsymbol{G}}_1=\frac{\left(\boldsymbol{M}-2\boldsymbol{P}\right){\left(\boldsymbol{M}-2\boldsymbol{P}\right)}^{\hbox{'}}}{2{\displaystyle \sum {p}_i}\left(1-{p}_i\right)}, $$

where **M** is a matrix of counts of the allele labelled ‘A’ (animals by SNPs), p_i_ is the A allele frequency of the *i*^th^ SNP, **P** is a matrix (with dimensions number of animals by number of SNPs) with each row containing the p_i_, **I** is the identity matrix (of size number of animals), and the ratio ${\sigma}_a^2/\left({\sigma}_a^2+{\sigma}_e^2\right)$ is the ‘heritability’ (h^2^) of the breed proportion. The allele frequencies were estimated from all samples available in October 2010. Missing values in **M** were replaced by two times the breed A allele frequency (weighted by breed proportion, and including a breed group for breeds other than the prediction breeds considered here). The variances are nuisance parameters to be estimated, possibly with a constraint to a fixed h^2^. The data were analysed either using h^2^ fixed at 0.95 (‘fixed h^2^’ analysis), or without constraint on h^2^ (‘estimated h^2^’ analysis). For the ‘fixed h^2^’ analysis, h^2^ was fixed at a high value (0.95) as recorded breed proportions would be expected to be inherited exactly (as the parent average), but may not be due to recording error, rounding, or differences between genomic relatedness and pedigree relatedness. “Model-based accuracies” were calculated using BLUP methodology [19].

For animals not in the training set, breed proportion mBVs can be calculated by including them in the above analysis, but with missing breed data, or by using equations from VanRaden [18], which allow the mBV to be calculated directly as a linear function of the animal’s SNP (0/1/2 scored) data.

### Validation

The equations were applied to animals not used in training. In the first instance, the remaining animals from the October 2010 dataset, and of the breed types used in training, were used. There were 237 Romneys, 205 Coopworths, 18 Perendales, 16 Texels and 42 CompRCPs in this group. Subsequently the equations were applied to all 13,118 samples with genotypes (8,705 of which had recorded breed available from SIL), 4,318 of which were in the training set. A few training samples were no longer identified in this more recent dataset, due to ongoing data edits possibly including animal identifier corrections.

### Regression method

Prediction equations were also developed using the ‘regression method’ of Kuehn *et al.* [7]. In this method

$$ \mathbf{y}\sim \mathbf{X}\mathbf{b}+\mathbf{e} $$

where **y** is a vector (of length the number of SNPs) of proportion of A alleles in the genotype for each SNP (i.e. half the number of A alleles) for the animal in question, **X** is a matrix (with dimensions number of SNPs by number of prediction breeds) of allele frequencies for each breed, **b** is a vector (of length number of prediction breeds) of the proportions of each breed in the animal (to be estimated) and **e** is a vector of residuals (of length number of SNPs). This method was applied with four prediction breeds (Romney, Coopworth, Perendale and Texel). The set of breeds used will have some influence on the results, unlike the genomic selection method where each breed prediction equation was found independently. Breed allele frequencies were calculated from the subset of pure breed animals (i.e. recorded as 100% of the relevant breed) for use with the regression method (denoted ‘Regp’ to emphasise that pure breeds were used). There were 2445 Romneys, 479 Coopworths, 281 Perendales and 36 Texels in this pure-breed subset.

### STRUCTURE method

Predicted breed composition was calculated using version 2.3.4 of the program STRUCTURE [4]. The pure-breed training animals (the same set as used to calculate breed allele frequencies for the Regp method) were designated as being from a known population (breed). Due to memory limitations, only the subset of animals available in October 2010 were analysed with STRUCTURE. In addition, it was necessary to reduce the marker set to 10,000 SNPs. Three different 10,000 SNP sets were investigated, two where the SNPs were chosen at random, and one where the top 10,000 SNPs for breed assignment informativeness, using equation (4) of [20], were chosen. Results from different marker sets were all very similar, except for the probabilities of some of the training animals belonging to their designated population. Analyses were run using either 4 or 5 populations, but results were very similar, with the highest proportion of an animal belonging to the additional (non-training) population being 0.04. Only the results from the markers chosen on informativeness and using 4 populations are presented here. Results are presented for a run with a burn-in of 10,000 and 10,000 subsequent replicates; results were highly consistent with those from an independent run using a burn-in of 1000 and 1000 replicates (these smaller numbers were also found to be adequate for consistent results when using other marker sets).

### Principal components analysis (PCA)

Principal components were also calculated, both as a method for understanding breed composition, and for graphically displaying data and results. Principal components were calculated from the genomic relationship matrix (G_1_; discussed above) using the prcomp function of R [21].

### Detection of breed errors

A metric was calculated for each of the methods to index the discrepancy between predicted breed and recorded breed. For the GS and Reg methods, predictions were truncated to be within the feasible range (0 to 1), and the metric calculated as

$$ Er{r}_{method}=\sqrt{{\displaystyle {\sum}_b{\left( predicted- recorded\right)}^2}} $$

where *b*∈{Romney, Coopworth, Perendale, Texel}.

A PCA based metric was calculated as the standardised Euclidean distance from the breed mean using the first 10 principal components (PC1-PC10) as follows. Each animal from the full set of 13,118 animals was initially classed as either a purebred (recorded as 100% of either Romney, Coopworth, Perendale or Texel) or ‘Other’ (including those without a recorded breed). Breed means (*m*_*kb*_) for the *k*th PC (*k* = 1,…,10) were found for each of these groups (*b*∈{ Romney, Coopworth, Perendale, Texel, Other}). For example, *m*_1Coopworth_ = −1.26, *m*_2Coopworth_ = −3.00, which can be seen to be central to the Coopworth region in Figure 2. Then for an animal *j* with recorded breed proportions of π_*jb*_ (using ‘Other’ to denote any recorded breed other than Romney, Coopworth, Perendale or Texel or the breed for an animal not on SIL) the distance from its breed composition mean was calculated as

$$ {d}_j=\sqrt{{{\displaystyle \sum_k\left(P{C}_{jk}-{\displaystyle {\sum}_{\mathrm{b}}{\pi}_{jb}}{m}_{kb}\right)}}^2} $$

where PC_*jk*_ is the value of the *k*^th^ PC for the *j*^th^ animal. The standard deviations (*s*_*b*_) of these within each of the pure breed groups was found, and the final metric was calculated as

$$ Er{r}_{PCA,j}={d}_j/{\displaystyle {\sum}_{\mathrm{b}}{\pi}_{jb}{S}_b} $$

## Additional files

Additional file 1:
**Number of animals by year of birth and breed.** Plot of the number of animals in the study by year of birth and breed. Additional file 2:
**SIL-recorded breed statistics for the 8,705 SIL recorded animals.** Number of animals with some proportion of the breed, the sum of the breed proportions across all animals, percentage of the resource and average proportion of a breed in animals containing that breed.
