# Supplementary material for: Genomic breed prediction in New Zealand sheep
Source: BMC Genet. 2014 Sep 16;15:92. doi: 10.1186/s12863-014-0092-9 (PMC4353690; doi:10.1186/s12863-014-0092-9)
Supplement: Additional file 1: — Number of animals by year of birth and breed. Plot of the number of animals in the study by year of birth and breed. [file 12863_2014_92_MOESM1_ESM.docx]

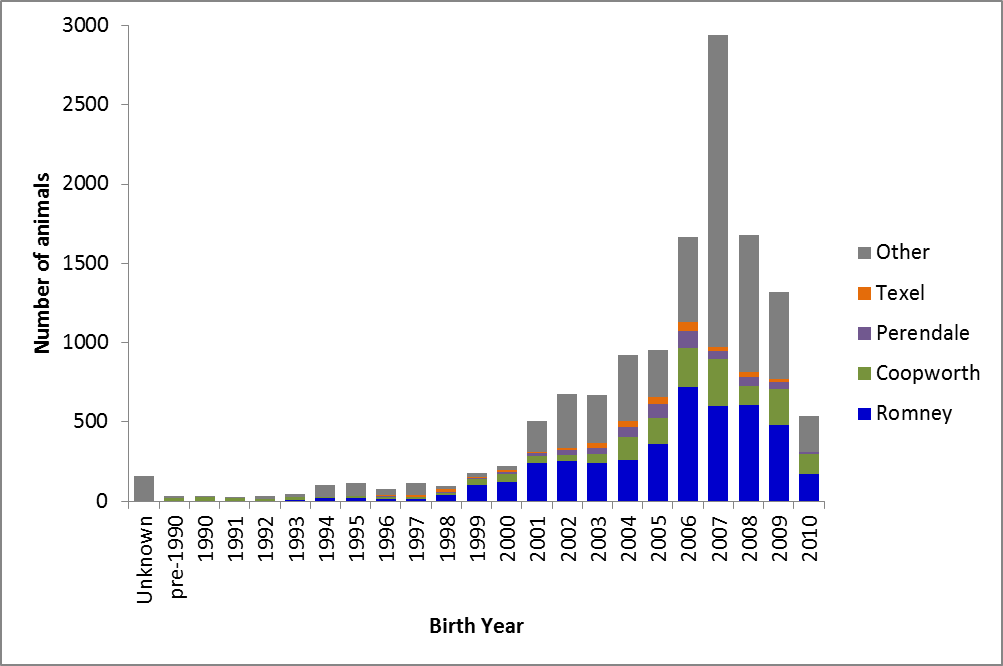


**Figure S1 - Number of animals in the study by year of birth and breed.**

Animals are denoted as their major breed if this is at least 75%. ‘Other’ includes animals of other breeds, breed composites and animals not recorded on SIL.
