# Supplementary material for: Genomic breed prediction in New Zealand sheep
Source: BMC Genet. 2014 Sep 16;15:92. doi: 10.1186/s12863-014-0092-9 (PMC4353690; doi:10.1186/s12863-014-0092-9)
Supplement: Additional file 2: — SIL-recorded breed statistics for the 8,705 SIL recorded animals. Number of animals with some proportion of the breed, the sum of the breed proportions across all animals, percentage of the resource and average proportion of a breed in animals containing that breed. [file 12863_2014_92_MOESM2_ESM.docx]

**Table S1 SIL-recorded breed statistics for the 8,705 SIL recorded animals.**

| Breed name | Number of animals with breed | Full breed equivalent | % of total resource | Average proportion in animal |
| --- | --- | --- | --- | --- |
| Romney | 5478 | 4544.9 | 52.0% | **0.83** |
| Coopworth | 2999 | 2029.4 | 23.2% | **0.68** |
| Perendale | 800 | 636.4 | 7.3% | **0.80** |
| Texel | 1743 | 626.1 | 7.2% | **0.36** |
| East Friesian | 1820 | 214.5 | 2.5% | **0.12** |
| Poll Dorset | 858 | 163.1 | 1.9% | **0.19** |
| Composite | 386 | 97.3 | 1.1% | **0.25** |
| Finnish Landrace | 535 | 75.6 | 0.9% | **0.14** |
| Romney Dorset | 220 | 69.2 | 0.8% | **0.31** |
| Suffolk | 94 | 60.4 | 0.7% | **0.64** |
| Corriedale | 87 | 58.1 | 0.7% | **0.67** |
| Lamb Supreme | 212 | 52.8 | 0.6% | **0.25** |
| Marshall Romney | 46 | 22.2 | 0.3% | **0.48** |
| Dorper White | 25 | 18.2 | 0.2% | **0.73** |
| Wiltshire | 35 | 9.6 | 0.1% | **0.28** |
| Dorset Down | 25 | 8.2 | 0.1% | **0.33** |
| Cheviot | 12 | 7.7 | 0.1% | **0.64** |
| Ile-de-France | 14 | 7.0 | 0.1% | **0.50** |
| Southdown | 6 | 6.0 | 0.1% | **1.00** |
| South Suffolk | 6 | 4.6 | 0.1% | **0.76** |
| Growbulk | 12 | 4.5 | 0.1% | **0.38** |
| Finn x Texel | 5 | 4.4 | 0.1% | **0.88** |
| Dorset Horn | 11 | 3.9 | 0.0% | **0.35** |
| Dorper Black | 3 | 3.0 | 0.0% | **1.00** |
| TEFRom | 5 | 2.5 | 0.0% | **0.50** |
| Texel Cross | 13 | 2.5 | 0.0% | **0.19** |
| Oxford Down | 25 | 2.0 | 0.0% | **0.08** |
| Hampshire | 2 | 2.0 | 0.0% | **1.00** |
| Primera | 2 | 2.0 | 0.0% | **1.00** |
| Highlander | 1 | 1.0 | 0.0% | **1.00** |
| Borderdale | 13 | 0.9 | 0.0% | **0.07** |
| South Dorset | 4 | 0.8 | 0.0% | **0.19** |
| Coopdale | 6 | 0.6 | 0.0% | **0.10** |
| Aust. White Suffolk | 11 | 0.6 | 0.0% | **0.05** |
| Ranger | 1 | 0.5 | 0.0% | **0.50** |
| Wiltshire Dorset | 12 | 0.3 | 0.0% | **0.02** |
| Landmark | 1 | 0.3 | 0.0% | **0.25** |
| Finnish Landrace Cross | 1 | 0.3 | 0.0% | **0.25** |
| Border Leicester | 1 | 0.1 | 0.0% | **0.13** |
| Lincoln | 1 | 0.0 | 0.0% | **0.02** |

“Full breed equivalent” is the sum of the proportion across all animals, “Average % in animal” relates only to animals with some recorded proportion of that breed.
